# Fog Computing Enabling Industrial Internet of Things: State-of-the-Art and Research Challenges

**DOI:** 10.3390/s19214807

**Published:** 2019-11-05

**Authors:** Rabeea Basir, Saad Qaisar, Mudassar Ali, Monther Aldwairi, Muhammad Ikram Ashraf, Aamir Mahmood, Mikael Gidlund

**Affiliations:** 1 School of Electrical Engineering and Computer Science, National University of Science and Technology, Islamabad 44000, Pakistan or rabeeabasir@gmail.com (R.B.); saad.qaisar@seecs.edu.pk (S.Q.); 2 Department of Telecommunication Engineering, University of Engineering and Technology, Taxila 47050, Pakistan; 3 College of Technological Innovation, Zayed University, Abu Dhabi 144534, UAE; monther.aldwairi@zu.ac.ae; 4 Centre for Wireless Communication, University of Oulu, 90014 Oulu, Finland or; 5 Department of Information Systems and Technology, Mid Sweden University, 85170 Sundsvall, Sweden; aamir.mahmood@miun.se (A.M.); mikael.gidlund@miun.se (M.G.)

**Keywords:** Industry 4.0, Internet of Things, Industrial Internet of Things, Cyber Physical System, cloud computing, fog computing, edge computing, smart devices, smart factory, industrial automation

## Abstract

Industry is going through a transformation phase, enabling automation and data exchange in manufacturing technologies and processes, and this transformation is called Industry 4.0. Industrial Internet-of-Things (IIoT) applications require real-time processing, near-by storage, ultra-low latency, reliability and high data rate, all of which can be satisfied by fog computing architecture. With smart devices expected to grow exponentially, the need for an optimized fog computing architecture and protocols is crucial. Therein, efficient, intelligent and decentralized solutions are required to ensure real-time connectivity, reliability and green communication. In this paper, we provide a comprehensive review of methods and techniques in fog computing. Our focus is on fog infrastructure and protocols in the context of IIoT applications. This article has two main research areas: In the first half, we discuss the history of industrial revolution, application areas of IIoT followed by key enabling technologies that act as building blocks for industrial transformation. In the second half, we focus on fog computing, providing solutions to critical challenges and as an enabler for IIoT application domains. Finally, open research challenges are discussed to enlighten fog computing aspects in different fields and technologies.

## 1. Introduction

Revolution in any realm is required with the passage of time. Every field changes to go forward with better solutions dealing with the challenges of the era. Industrial Internet of Things (IIoT) is revolutionizing the classical communication methodologies. With the emergence of smart devices (mobile, machines, sensors) coupled with a diverse range of applications requirements, IIoT is the way forward. It is expected that 26 billion IoT devices of heterogeneous capabilities will be installed to perform functions with different Quality-of-Service (QoS) requirements by 2020 [1]. IIoT gives rise to 4th industrial revolution based on Cyber-Physical Systems (CPS) with the need arising back in 2015 originated basically in Germany [2]. Industry 4.0 defines diverse use cases ranging from interconnected digital technologies, CPS, Mobile Cloud Computing (MCC) and Internet of Things (IoT) for promoting the whole industry in terms of efficiency, effectiveness, supporting heterogeneous data, higher production, automation, and integrating knowledge [3]. These key enabling technologies have been deployed to some extent in industrial domains such as healthcare, transportation, smart cities, micro-grids, and smart factory. This trend gives rise to intelligent, distributed and self-organizing solutions to support these application domains.

Deploying industry 4.0 involves three-layer implementation; physical layer, network layer, and intelligent-application layer [4]. The physical layer comprises identification and location awareness entities i.e. actuators, sensors, and terminal devices; the network layer comprises of the development of a network that can support industrial automation, network can be cellular, indoor, cloud or private. Factory automation and coordination are processed on the application layer. Infrared (IR), Radio-Frequency Identification (RFID), Bluetooth, 6LoWPAN, IEEE 802.11 af, IEEE 802.11 a/b/n/ac for short range connectivity; Ultra-Wideband (UWB), cellular (2G, 3G, 4G, LTE-MTC, 5G), Sigfox, Long range (LoRa) for long range connectivity, are a few of the majorly used communication standards for IIoT [5,6].

The future of automation is based on decentralized intelligence in which all machines can communicate with one another to arrive at independent or consensus inference, called Machine-to-Machine (M2M) communication. These decentralized intelligent solutions play a vital role in industry 4.0 digital transformation. The decentralized solutions provide flexibility and quick decision assistance over centralized solutions. For M2M communication, 802.11ah technology has evolved in the recent past. Exchanging machine data demands real-time communication ensuring latency, security, reliability, bandwidth and privacy measures in all IIoT domains. To satisfy these critical requirements, there is a need to explore new enabling solutions that support these applications. In the future, 5G cellular technology will support such heterogeneous networks with massive number of IIoT devices. It is anticipated that future 5G networks not only provide flexibility but can optimize the usage of available resources of bandwidth, power, energy, connectivity to different applications at the same time [7].

In the last decade, computation and processing requirements of end users have increased exponentially. It has become increasingly challenging for designers to scale the processing and data storage capabilities for users within the given device size and battery constraints. To meet these growing requirements, researchers have come up with the solution to offload services to a centralized location known as the cloud. Cloud computing is an alternative for data computation, storage and management. It supports intensive computation and manages heterogeneous devices of next generation networks [8,9,10]. Additionally, cloud computing architecture involves the direct connection between devices and the cloud server. Practically, we are beginning to understand the connection between and the enormous number of IIoT devices and a single cloud server. However, cloud-based systems are unable to meet the requirement such as heavy data computation, real-time device control, security and management results in insufficient support of IIoT application requirements [11]. Considering a wide variety of IoT scenarios, some of the challenges [10,11,12,13,14,15,16] in cloud computing are listed below:

- Large distance between the cloud and edge devices causes propagation and transmission delays.
- Large computational load on a single cloud server causes processing and queuing delays.
- Increased number of smart devices has hindered meeting the bandwidth requirements.
- Enormous number of smart devices will bring scalability, speed, and computational issues.
- Wireless medium between cloud and smart devices brings resource management issues.
- Heterogeneity property of smart devices in terms of accessing technology will bring difficulty in handling at the cloud.
- Mobility of IoT devices bring service availability issues, cloud server may not be able to provide services due to network congestion and failure.
- Security is a very critical thread, as the cloud is exposed to the whole world over the public internet.
- Computing offloading every-time at cloud causes a loss in energy and battery lifetime.
- Although data storage at cloud brings benefits to application developers, they should be careful of integrity and authentication demands of IIoT applications.
- Cloud computing is a centralized and complex architecture for real-time applications of IIoT.

All these limitations require a change, how and where we process data. These challenges motivate us to explore new decentralized approaches/solutions in IIoT domains. A new concept of fog computing is introduced by Bonomi et al. [15] for handling data locally at the network edge in order to overcome the limitations of cloud architecture. Fog computing complements existing cloud architecture and has addressed the issue of latency and bandwidth efficiency [17]. Because of its distributed architecture, it calls for a strong check on QoS requirements to make it useful. Fog is mainly based on distributed networking with ubiquitous pervasive computing. It comprises small scale data centers or a group of computers known as cloudlets (fog clouds) that provide services to devices located in close proximity [17,18]. The initial installation cost, latency, and energy consumption is far less as compared to that of the cloud, but the operational cost varies. Fog architecture can leverage computations either from dedicated edge servers or adhoc infrastructure. For promoting IIoT architecture with fog computing as a key enabler technology, a group of fog clouds can also be used.

In fog computing, data processing in single server (fog cloud) helps in achieving real-time and reliable communication. It puts the safety and security of personal data back into our premises. Furthermore, a cost effective approach can be used in fog computing such that data transmission and storage fees can be reduced based on service premises. Therefore, fog computing has the potential to provide affordable solutions for large IIoT projects. Instead of being restricted to only one expensive cloud connection, fog computing gives the freedom to choose any hardware from Information Technology (IT) solutions. It supports all existing legacy devices and non-IIoT devices that never intended to be the part of IIoT application. This is not only economical but also more flexible. When it comes to speed, fog computing allows real-time processing and supports to process data as fast as our local system. Fog can be managed securely from remote places. It can be scaled and updated dynamically. It gives more security, better performance, and lower costs. Fog incorporates positive attributes of cloud and provides benefits that may support future IIoT applications [18,19,20,21,22,23].

**Figure 1:**
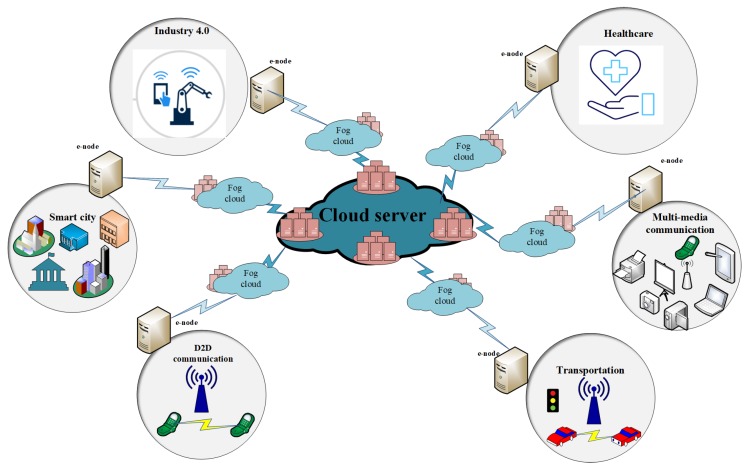
Generalized view: IIoT application domains with cloud, fog and edge computing.

Fog computing and edge computing being extended form of cloud computing gives solutions to the challenges faced by cloud computing that is attractive for IIoT real-time applications. The terms fog computing and edge computing are often used by industry interchangeably. Both these computing technologies bring computing and processing capabilities near the vicinity where data originates. Edge computing complements fog computing by bringing computation to one of the devices of a network. This device is named as E-node and is close to the data. E-node has more power, computation capabilities and intelligent controllers, such as programmable automation controllers (PAC). Presence of E-node in edge computing improves latency, reliability, security and privacy issues [24,25]. E-node acts as an interface/bridge between the data sources and the cloud. The basic architecture for fog network is given in Figure 1 depicting fog cloud serving as a middle layer between the cloud server and smart end-devices. Figure 1 demonstrates a basic idea of cloud, fog and edge computing promoting different IIoT application domains.

Fog is a relatively new paradigm that brings new challenges in terms of efficient and scalable network architecture. It is expected that it will gradually develop over the next few years for realizing the Industry 4.0. Challenges, such as energy conservation, real-time communication, efficient spectrum use, cache memory on edge devices and optimized allocation of resources are open issues that need to be addressed for future automation. Without such considerations, guaranteed QoS requirements of IoT devices may not be fulfilled. In the future, solutions to these challenges must be provided by researchers for the development of the industrial revolution. This paper is written with an aim to give a summarized version of existing solutions using fog computing acting as an enabler for IIoT applications.

**Figure 2:**
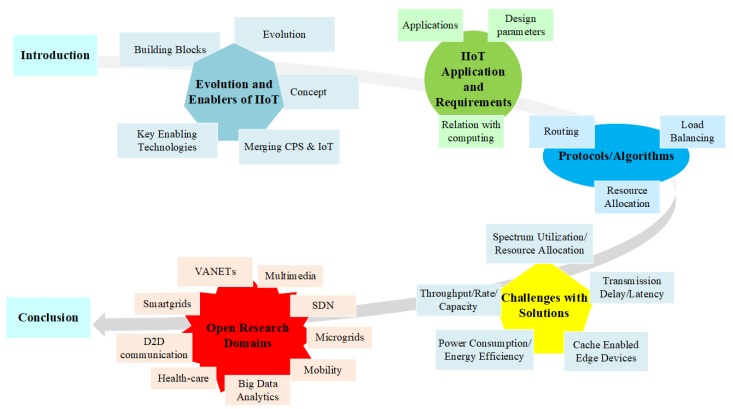
Flow of the paper.

The paper is organized as follows; Section 2 briefly introduces IIoT. Benefits of IIoT applications in daily life and their critical requirements are briefly explained in Section 3. Section 4 presents protocol/solution proposed by various researchers promoting fog computing as an enabling technology for IIoT development. Section 5 describes challenges and solutions in communication and networking proposed in the literature to use fog computing in IIoT. Section 6 lists down several open research issues in fog computing. Finally, the paper is concluded in Section 7. The flow of this survey paper is shown in Figure 2.

## 2. Evolution and Enablers of Industrial Internet of Things

As discussed earlier, IIoT or Industry 4.0 is a new emerging term for future industry, which involves many key enabling technologies and applications of IoT. In this section, Industry 4.0 evolutional phases, IoT connectivity technologies, benefits from IIoT and key enabling technologies that endorse industrial revolution are briefly explained.

### 2.1. Industry 4.0-Evolution

**Figure 3:**
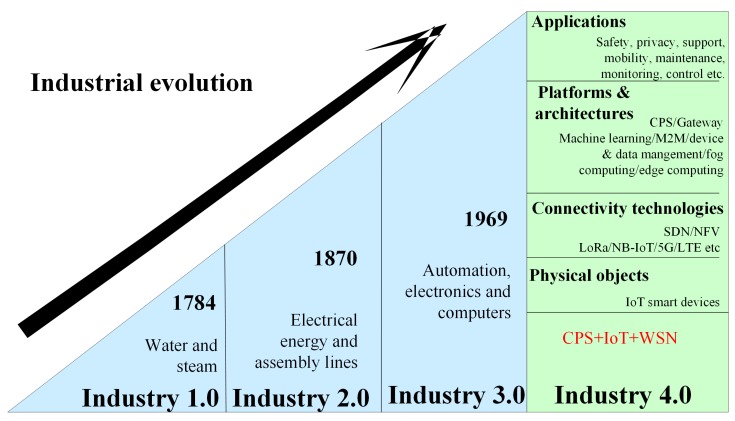
Evolution towards Industry 4.0/IIoT.

The industrial revolution with the passage of time has many phases according to the requirement and challenges of the respective era. Figure 3 gives an idea about evolution towards Industry 4.0 with its elements.

**Industry 1.0:** At the end of the 18th century, the 1st industrial revolution started with the help of water and steam power, which systematizes the factory floor. First, the mechanical weaving loom was established in 1784, and the first mechanical system was built thorough mechanical production facilities.

**Industry 2.0:** In the beginning of the 20th century, the 2nd industrial revolution started using electrical energy. The first assembly line using electrical energy was established in 1870. The introduction of mass production in industry 2.0 enhanced the industry.

**Industry 3.0:** Beginning of the 1970s i.e., in 1969, the first control system using programming language was established. Industry was slowly shifted to automation using information technology and micro-electronics’s applications. This is the 3rd industrial revolution [26].

**Industry 4.0:** This previous industrial revolutions give rise to the development of industry 4.0. Industry 4.0 contributes a revolution to all domains comprising economic, academic, research, industrial and manufacturing sectors. There is a huge impact of the industrial revolution on the manufacturing processes of many fields. Implementing industry 4.0 demands change in many technologies namely automation, identification, computer, network communication, digital manufacturing, production process, production control management, decision making, judgment, sensing and analysis [27]. In the future, the manufacturing industry is expected to change on a large scale because of all new generation networks and interfaces offered by the environment of industry 4.0. This transformation is already in process in many industrial sectors. Up till now, for the fourth industrial revolution, exponentially growing technologies are sensor technology, artificial intelligence, machine learning, robotics, nanotechnology, and 3D printing [28]. These technologies were invented decades ago, but their minimum cost and exponential growth will shape industry 4.0. To change the industrial process, researchers are focusing on providing the evolved form of these technologies in terms of flexibility and fast computational process. All this automation in industry is very important for the economic growth of a country.

### 2.2. Industry 4.0-Concept

Increasing progress is witnessed in automation of industry using advancement in digitization, networking and new communication technologies, satisfying the market and consumer requirements [29]. Lenze SE, Corporate Communications Public Relations use idea of *machine modularization*. According to the demand of market/consumers, different modules are added or removed during the manufacturing process; machines are retooled smartly using smart communication technologies A cloud solution was given by Lenze, which is secure as no customer wants to share their demands and production details. Details about the customer’s machine are saved in a cloud and he can investigate all the system details especially faults. This cloud solution is vulnerable to hackers, Lenze, along with another company, have provided a secure solution that is acceptable for today’s industry [30].

Charlotta Johnsson explained the idea of industry 4.0 using four terms, these are smart devices and smart production processes with horizontally and vertically integrated manufacturing systems. Smart devices result in the production of intelligent products, these products do self-monitoring, self-controlling and self-manufacturing, have a uniquely identifiable ID, know how to solve and achieve goals [31]. The intelligent production process comprises smart starting and ending of manufacturing processes. Vertical and horizontal integration means all the steps during the smart/intelligent production process are integrated throughout the life cycle i.e., from starting phase to ending phase [30].

Industry 4.0 results in a faster manufacturing process, product development and improves the handling of complex environments inside an industry. The term first originated in Germany named as *Industrie 4.0*; in United States term used for this fourth generation is *Smart Manufacturing*, Chinese researchers have used term *China 2020*. *Industrial Digitisation* is the term used in Sweden for transformation of industry to automation [32]. It is believed that this industrial revolution will increase global competitiveness, preserve the domestic manufacturing industry and will have a huge impact on the business market as well. Now many countries around the globe have taken initiatives for automation in industries.

### 2.3. Industry 4.0-Merging CPS and IoT

**Figure 4:**
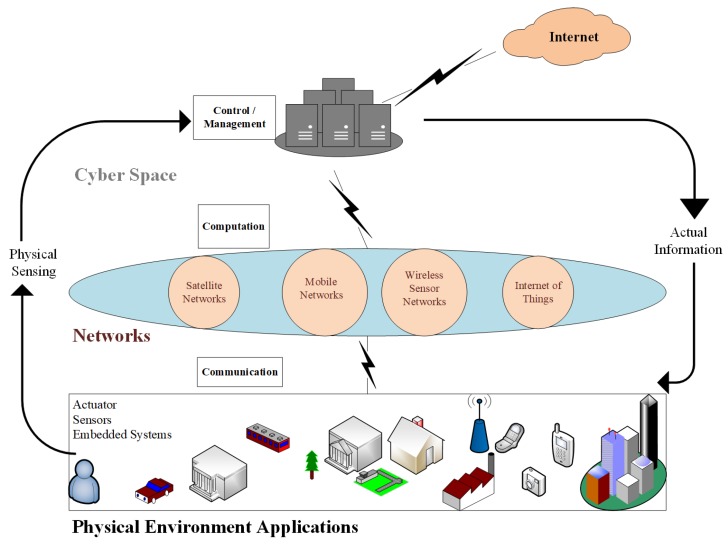
A cyber physical system architecture.

**Figure 5:**
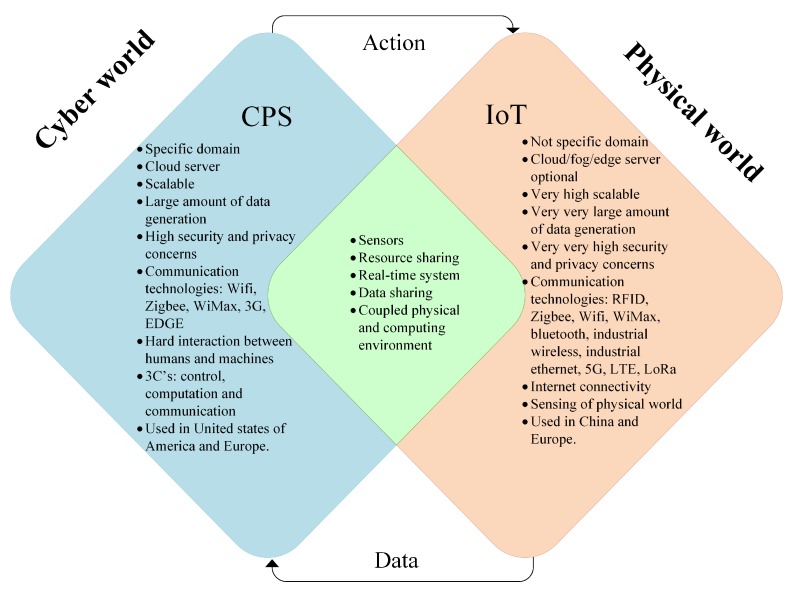
Comparison of CPS and IoT; supporting Industry 4.0 development.

The Industry 4.0 environment is comprised of the Internet of data, Internet of things, Internet of people and Internet of services. Interface of Industry 4.0 with existing smart infrastructure such as smart buildings, smart homes, smart grids, smart logistics, social web, and business web build a CPS system. This revolution will merge the real and virtual world on the basis of CPS. A CPS system has a computer-based algorithm that integrates the Internet and its users. It is simply digitization, in which these systems make connection of information technology with electronic/mechanical device components that exchange information among each other using a network. Using computer-based algorithms, CPS brings software and hardware components working in an automated and controlled manner to perform a certain task without human’s assistance. The basic visualization of a CPS is given in Figure 4. After collection and analysis of big data, CPS can increase performance in terms of high-quality, low-cost goods production. With the advancement in sensors and computing technologies, various CPSs are emerging. CPS has evolved to Cyber Physical Production Systems (CPPS) to encourage the development and production process of Industry 4.0 [33]. CPPS combines physical smart IoT devices, networking technologies to compute in the production process. Robotics, remote machinery control and diagnosis, smart devices, heavy industry, transportation, health and condition monitoring, energy production, smart cities, and food manufacturing are IIoT services enable by CPS/CPPS architecture. Figure 5 represents the comparative analysis between CPS and IoT in the form of a Venn diagram. The similarities between the two give support for the development of Industry 4.0/IIoT.

The layered architecture of Industry 4.0 is given in Figure 3, which involves the common attributes of CPS and IoT. The physical or sensing layer should be designed to the extent that IIoT applications can sense/control information from physical environment and integrates with hardware sensors and actuators accordingly. The second layer should be optimized to provide a reliable connection to support data transfer over a communication medium (wired/wireless). So far, IIoT applications used wired medium to provide solutions. In the near future, the wireless medium is required because of shifting from centralized to decentralized solutions. Connectivity technologies, such as NB-IoT/5G and beyond 5G over different architecture such as SDN, NFV, cloud computing or fog computing will give solution to different applications. The intelligent-application layer, providing services to users has to be optimized in terms of service production, satisfaction, interaction, and management.

### 2.4. Industry 4.0-Key Enabling Technologies

**Figure 6:**
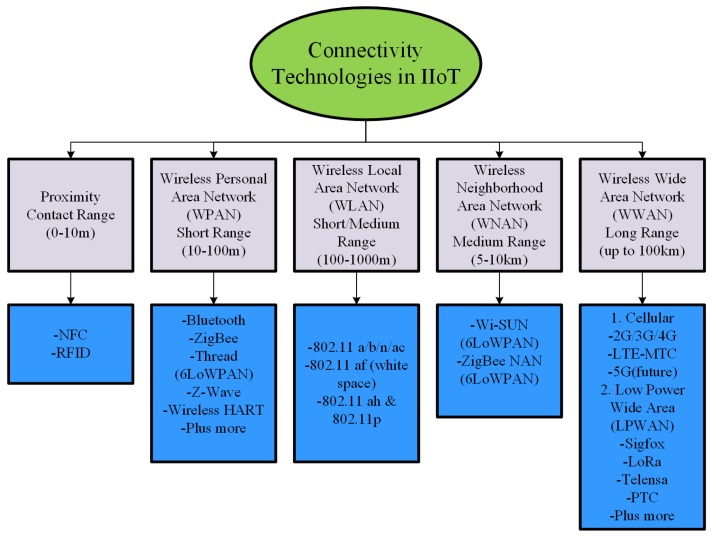
Different connectivity technologies in IIoT.

With the use of advance technologies of wireless communication, diverse new emerging protocols and architectures are supporting automated industry 4.0 development. Resources can be efficiently used after integration of communication technology and big data processing in real time, this will result in better performance. Industry 4.0 development involves many communication technologies; however, big data, IoT, 5G, mobile computing and cloud/fog/edge computing are the key enabling technologies [2,34,35]. An extensive range of IIoT projects have been deployed in domains of building automation, manufacturing systems, health care systems, transportation systems, processing food and agricultural systems in the past few years. Reaching a common task in an IIoT application; sensing, integration, and communication are main steps. RFID tags are used for sensing, network topologies and protocols are used for communication. All these smart devices are associated with each other using internet. Many connectivity technologies are available for supporting IIoT applications. Critical requirements of IIoT applications have many open challenges in all domains (smart grid, smart cities, smart devices, D2D, healthcare) such as capacity, real-time connectivity, remote maintenance and topology of communication networks. Figure 6 gives a general overview of technologies to connect things to the Internet, representing short-range and long-range wireless technologies. All technologies work differently with aim of low-latency, low-power consumption, low-bandwidth requirement, and reliable communication.

For connecting IIoT devices, all technologies have to work with the objective of maximum throughput, minimum power consumption, minimum transmission delay, and maximum transmission distance range. 2G, 3G, 4G, LTE are cellular technologies that were used for long range connectivity in wireless wide area networks (WWAN). IIoT application’s critical requirements and the increasing number of smart devices need additional resources for connectivity. Increase in smart devices results in more data processing for which connectivity technology is moving towards 5G. The 3rd Generation Partnership Project (3GPP) proposed Extended Coverage-Global System for Mobile Communications for the Internet of Things (EC-GSM-IoT) and Narrowband-Internet of Things (NB-IoT) for supporting M2M, enhanced MTC (eMTC), massive MTC (mMTC) and critical MTC (cMTC) communication networks for IIoT applications [5]. 5G cellular technology gives super low latency, ultra-reliable and high availability to cMTC applications (industrial application and control, remote surgery, remote training, remote manufacturing, and traffic safety and control). Low cost, low energy, and the massive number of intelligent devices in smart agriculture, smart meter, tracking, fleet management, and logistics domain are supported by 5G as well. 5G is beneficial for IIoT applications comprising from mMTC, cMTC to enhanced mobile broadband. The distributed model of IIoT applications require a massive amount of data rate with minimum latency, 5G technology gives 10 Gbps with 1 ms latency. 5G is use case driven communication technology for upcoming IIoT applications.

For distributed ultra-low-latency and reliable connectivity in IIoT applications, 5G-IoT is an emerging solution. 5G-IoT scenario extends capabilities of IoT smart devices used in all domains. Recent research is focusing on low-latency, end-to-end reliability, and low energy consumption for both uplink and downlink communication. There is a lot of potential in research on IIoT with 5G communication technologies, to overcome challenges. This research will help in the industrial revolution. With the evolution of Industry 4.0, 5G is rapidly evolving in order to meet the requirement of IIoT applications mainly real time functioning, energy efficiency, less power consumption, shared spectrum regulation, reliable communication, and handing massive amount of data. Almost 90% of needs met using fixed line 3G and 4G cellular technologies, but need for deployment of industrial revolution can be fulfilled using 5G. 5G as an enabler of industry 4.0 gives multi-channel, capability, multi-network management, operating both local and global networks, supporting heterogeneous networks [27]. Mobile computing and cloud computing brings accurate data for IIoT application and provide efficiency to industry 4.0 infrastructure. Details of cloud computing in comparison with fog and edge computing is explained in the next section.

### 2.5. Industry 4.0-Building Blocks

Fourth manufacturing revolution, i.e., digital industrial technology provides services in industries that involves data exchange among machines making more efficient and fast processes. In words, the IIoT can be defined as: *Devices with centralized controllers, sensors, battery and memory attributes will interact with each other using Artificial Intelligence (AI) and Machine Learning (ML) algorithms.* Real time connection is possible using decentralized analytics and decision making of these devices. This section will give building blocks that are used in transforming industry 4.0 development.

#### 2.5.1. Simulation, Autonomous Robots

A virtual model of a physical world which comprises machines, humans, and products can be interpreted in real time technology named as simulation. Every new product or updating process in available products for any machine can be verified, tested and optimized via simulation-based applications. It will result in increasing the quality of machinery and save the resources in the physical world. An autonomous robot collects information from its environment and learns from it and does work in the future without the involvement of humans using its self-learning algorithms (machine learning). These robots will transform the industries into automated industry. This technology will have a large impact on the industrial revolution. These robots are cheap and more capable of doing tasks efficiently.

#### 2.5.2. Big Data and Analytics, Horizontal and Vertical System Integration

Different systems, ranging from customer to enterprise-level systems, have to collect, manage and evaluate the big amount of data. Three main goals of big data analytics result in the reduction of cost, efficient decision making and emerging new services and products. Industry 4.0 involves digital transformation in vertical and horizontal value chain networks. These networks result in the integration of customer and enterprise systems of companies, departments and business market-exchanging data. These value chain processes should be transparent and flexible with real-time functioning constraint.

#### 2.5.3. Additive Manufacturing

Additive manufacturing is the process in which a 3D model is manufactured by joining the raw materials, usually layer by layer. It is the opposite of subtractive manufacturing in which raw material is carved to create a 3D model.

#### 2.5.4. Augmented Reality

The idea of taking decisions remotely in real-time results in improving work procedures and will be implemented in the future as Augmented Reality (AR). In this augmented reality-based systems send repairing requirements or selection of new components.

#### 2.5.5. Cyber-Security

Exponential increase in connections among devices in industry 4.0 will increase threats to systems, networks, and processes. Cyber-security is a process that prevents unwanted intruders from accessing, destroying, interrupting or changing sensitive information about company/organization as well as business market networks; gives them secure and reliable communication systems.

#### 2.5.6. Cloud Computing

Cloud computing is centralized and complex technology that supports high speed, high performance, flexible resource use and dynamic allocation in a network. As IIoT application requirements are low latency, high speed and reliable communication, privacy and security, efficient allocation of resources and energy-efficient communication technology. Some limitations regarding use of cloud computing for IIoT applications are:

- Confidential data and personal information of an industry should not be shared with outsiders.
- Security and privacy are in high demand by an industry from the cloud service provider.
- Data location on the basis of geographic follows rules and regulations. It also helps in securing the information.
- High load demands high-speed internet connectivity. This processing causes delays in communication.
- Memory and storage capacity may get exhausted because of many applications simultaneously accessing a single cloud server.
- Context awareness is required for speedy processes.
- Different standards cause problems in exchanging data, information, services, and applications among different clouds at different locations.
- Recovery and back-up update are required for industrial processing and decision making, cloud computing will cause delay.

#### 2.5.7. Fog Computing

Fog computing or “fogging” is an extended form of cloud computing, in respect of industrial revolution giving applications and services (low latency and high processing) to autonomous heterogeneous devices inside an industry [36]. The idea is to bring processing, storage, maintenance and intelligence control to the proximity of data devices. Inside industry 4.0, there is critical requirement of real-time services with high data processing, maximum capacity and scalability. Fog computing gives the best solutions for such an environment because of its significant benefits over cloud computing. Extension of cloud computing, aims to minimize the burden on the cloud by introducing network edge computing concept.

For industrial automation, real-time services and decision making processes require low latency and enhanced cache memory. Required performance parameters are mobility, real time applications, low-latency, location-awareness, number of nodes and cache-enabled edge devices on this basis of geographical distribution. Virtualized nodes frequently known as cloudlets or fog nodes are placed between clouds of internet and end user devices. Fog computing provides services and applications as a cloud does with better QoS parameters performance covering critical requirements of IIoT. Important advantages of fog computing that influence its use for IIoT are:

- Data storage on network edge nodes eliminates the transmission delay by removing the need for accessing data from far-away clouds.
- Fog computing supports to process and analyze the data on faster speed for IIoT applications.
- Data storage on edge nodes will reduce the processing and computing delay.
- Cache enabled nodes will prevent transmission of irrelevant information over the network.
- Can give support to all IoT applications e.g., smart grids, smart cities, D2D, Vehicular Ad-hoc networks (VANETS) using edge networking concept.
- Provides filtered and required interaction between end devices and cloud service providers.

Fog computing is the building stone to provide solutions for more efficient, effective and manageable communication way for the massive number of smart IoT devices in the near future. Fog computing with extra features as compared to cloud computing in terms of latency, security, location awareness, location, and number of server nodes, real-time connectivity and mobility is a promising enabler for industrial automation.

#### 2.5.8. Edge Computing

Introduction of enormous smart devices making an industrial revolution in all domains, causes extensive data processing, computation and burden of traffic on a single server either a cloud server or cloudlet. This motivates researchers to develop a new computing technology named as edge computing. The idea is to develop embedded automation controllers on devices named as edge-node (e-node) in the literature. This device is intelligent, with low processing power, better hardware security. Edge computing is an extending form of previous fog and cloud computing technologies. It comprises peer-to-peer networking, self-organizing network, and remotely manageable server. It gives following advantages:

- Encourages real-time connectivity.
- Overall network traffic reduces, as some computation is done on the edge of the network.
- Enhances security by encryption of data near to the network core.
- Optimize the resource usage.

IIoT applications have critical communication requirements. Cloud computing, fog computing, and edge computing platforms need to be optimized for better, efficient results. Cloud computing can be used where there is no high requirement of real-time connections, privacy, and security. On a local area network, fog computing uses a centralized system which interacts between the network and cloud server, whereas edge computing does computation on embedded systems of the network. Edge computing has direct interaction with sensors and actuators. The need for cloud, fog and edge computing architectures is increased with the growth of the IIoT application. To increase the use of IIoT smart devices, researchers are focusing on fog or edge computing paradigms which results in industrial development.

## 3. Industrial Internet of Things Applications and Requirements

Information about the occurrence of faults, components, inventory, different demands and different orders continuously needs to be shared among smart devices/processes resulting in improved efficiency, tracking, capacity use, quality of production and development in industries. From IIoT perspective; smart cities, smart factories, and smart products are important IIoT beneficial examples. The basic three-layer architecture of IoT as discussed in Section 2.3, need to be evolved according to the requirements of specific IIoT applications.

### 3.1. IIoT-Applications

From the identification of faults to solutions via communication and networking technologies, every step needs to be optimized and has research potential. Possessing attributes of intelligence, reliability, safety, sustainability, privacy, and efficiency; these applications are called *smart* in the literature. This revolution results in the development of new infrastructure. According to [37], smart city, smart factory and smart product are the main applications of industry 4.0.

#### 3.1.1. Smart City Applications

About 6 billion people are expected to be part of cities of the earth in 2050 [38]. With the advent of technology infrastructure, this increase in population will result in more data origination and demand for services. This big data origination is called *Big Data*. To develop future smart city, there are many domains that need to be intelligent, such as the smart home, smart office, smart institution, smart health-care centers, smart agriculture, and smart transportation. All these domains have different IIoT applications with different requirements. The development policy of a smart city has six factors namely, smart economy, smart mobility, smart environment, smart people, smart living, and smart governance [2]. Numerous research has been carried out in the domain of smart cities. For instance, [39] presents a framework with which, smart cities can overcome current limitations. This smart city transformation will take time.

#### 3.1.2. Smart Factory Applications

Smart industry comprises distributed automated systems and robotics. This future smart factory floor is possible using ML algorithms and AI technology. These automated devices are integrated with sensors, actuators, microchips, autonomous systems, and controllers. Relying upon CPS and IoT technologies for evolution industrial processes required ultra-reliable and low-latency communication (URLLC). For monitoring, managing and controlling such environment, IoT nodes can handle bounded latency of millisecond scale. These applications are characterized using latency, jitter, energy consumption, workload parameters. M2M and D2D are emerging supporting technologies for smart factory development. In the smart factory manufacturing process, machines will have high-level of automation and self-optimization attributes. These attributes will fulfill complex requirements of products. This has open issues regarding network communication technologies, number of devices, security, and cost.

#### 3.1.3. Smart Product Applications

IoT, cloud computing, big data, cloud computing and production time are drivers of industry 4.0 development. The products in industry 4.0 are smart because they are integrated with sensors and microchips. Existing production systems need to be integrated with industry 4.0 architecture (IoT+CPS+WSN). This integration will allow communication interaction between human beings and products [40].

### 3.2. IIoT Application Design Parameters

To the increasing demand of customers and market requirements, the manufacturing industry is now facing problems in achieving desired goals. Industry 4.0 came up with an innovative idea of automation inside in the industry increasing the production process flexibly. Industry 4.0 key point is M2M communication, in which machines communicate with each other over the Internet. Developing and manufacturing industry, communication of intelligent machines with each other using different technologies depending on the coverage area, give rise to high production and self-regulation of the manufacturing process in an industry. These are the goals of industry 4.0; all this is assured by IT systems as they provide enabling smart technologies. The integration of these heterogeneous devices in a network with other devices and existing communication technologies is also the main requirement for designers. Every IIoT application has critical design goal requirements to improve QoS in providing solutions. These design parameters are:

- **Energy & Long Battery Life:** Overall network energy should be preserved for better and efficient outcomes. Smart devices should have enough battery storage so that they can use for long time.
- **Latency:** Some IIoT applications are time-sensitive, a bound should be there to limit all types of delays including processing, propagation, transmission, and computation.
- **Throughput:** Amount of data for processing is different for different applications. It should satisfy the application requirement.
- **Network Topology:** How the number of servers (cloud, fog, e-node) and smart devices are placed in a network for better QoS requirements.
- **Reliability:** Solutions by IIoT applications demand reliable real-time connectivity.
- **Security, Safety & Privacy:** These are very demanding and major requirements for all IIoT applications. For example, inside a smart factory there should be privacy and security such that no one can access the private information. For healthcare applications, patient’s information should be safe and not easily accessible and changeable. 3A’s; Authentication, Access, and Authorization are steps involved in the strictly secure system. The demand of end to end communication in IIoT applications requires privacy of data as well. Sensors and actuators should be safe from intruders as well as environmental hazards.
- **Low Cost:** Smart devices used for IIoT applications should be low cost so that doesn’t affect the CAPEX/OPEX. Deployment involved in industry 4.0 should not be so much that will cause loss in marketplace.
- **Long Coverage:** A device should be capable enough to cover the desired range.
- **Standardization:** So far, there is no such network standardization and is an open challenge for researchers.
- **Integration:** IIoT applications are composed of heterogeneous devices and hybrid networks, there are a lot of issues in integration.
- **Communication/Enabling Technology:** Communication technology for supporting IIoT application should provide assured performance services.
- **Device Maintenance:** Heterogeneous device in an industry 4.0 environment, require constant device management as devices are connected with each other and the Internet. Software Defined Networking (SDN) is used for such failure and changing maintenance issues of devices.
- **Monitoring Network:** Wireless, environmental and mobility nature may cause a change in network topology which requires the system to be monitored and managed frequently.
- **Configuration & Management of System:** Self-configurable, self-control, reconfiguration functionality in addition of new devices in network.
- **Traffic congestion & Overload:** Smart devices will be increased with time in any IIoT application. System should be able to adjust according to the traffic burden and data requirement.
- **Mobility:** IIoT applications, such as transportation, inside industry and healthcare devices, have the property of mobility from one place to another.
- **Scalability:** Scalability brings many issues, some are: How many numbers of smart devices are enough to support an industrial application environment? or how many devices are served by a server easily? how to optimally design a system under energy/spectrum issues?
- **Heterogeneity & Interoperability:** Heterogeneous smart IIoT devices have to communicate and collect information among themselves and the Internet. This integration is an issue to solve. Standardization is required for interoperability of IIoT devices.
- **Performance:** There is always a performance trade-off among these QoS requirements. There should be an optimized, supportive, and efficient trade-off among the factors affecting performance. Performance maintenance solutions are required for future automation.

### 3.3. IIoT Applications in Relation to Cloud, Fog and Edge Computing

All IIoT applications require critical QoS parameters in order to produce benefits in every field. Fog term used in computer science is an extension of cloud architecture which offers services of cloud to edge devices. It is seen as a new cloud or it will replace the cloud in future but it is just an architecture that complements cloud architecture in order to provide solutions for critical applications. Entry points in any network are called edge devices. Entry points are part of second and third layer, it has hardware devices named as switches, routers and WAN devices. Cloud is a centralized solution and fog brings solutions at a distributed level by bringing data storage and its computation near the edge of a network. It allows getting services in the proximity of IoT devices. Fog combines services provided by cloud and IIoT applications; or it enables IIoT applications.

Cloud architecture is efficient, beneficial and provides solutions. It offers services after storing data in remote centers from the Internet. These remote data centers face less delay and computation as compared to internet. It saves the cost of physical resources, makes connections more reliable and results in an increase in efficiency and performance. It is a flexible, innovative framework in the networking field. It helps in accessing resources anywhere anytime. Question is why fog architecture is needed when cloud is already the best solution to many issues? There are certain issues and requirements of IoT applications and fog can provide solutions. First and foremost, the low-latency requirement of IIoT applications in every field can be attained effectively. It stores data in the proximity of users. Propagation delay between cloud and users will be reduced; computation delay due to huge data traffic at cloud can be reduced. It supports time-sensitive tasks effectively. Second IoT challenge was using network bandwidth in an optimized manner, fog being in the center of cloud servers and end devices. It helps in less usage of bandwidth; data doesn’t have to travel fog-to-cloud distance. Popular content is available at the network edge. This will also result in minimizing cost, lifespan of devices, energy consumption and complexity during every demand which goes on the users-to-cloud path. Security issues can be seen on the fog server; it can act as a proxy-server controller. Privacy and safety of data is another important IIoT devices requirement. Fog helps in monitoring such tasks.

**Figure 7:**
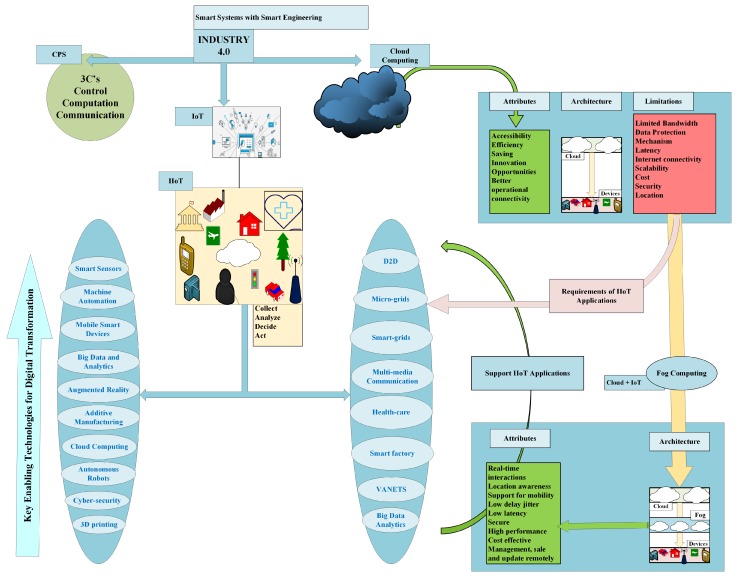
Industry 4.0, IIoT applications versus cloud and fog computing.

Exponential increase in IIoT applications in every field making world a cutting edge technology paradigm. Figure 7 shows both these architectures; cloud computing in which IoT devices are directly connected to data centers and cloud server and fog computing in which fog server is in the middle of cloud and IoT devices. Fog works on network edge which improves speed, computing capabilities and provides distributed and better solutions. Fog has complemented the cloud architecture in many ways. Cloud is a centralized solution while fog can work in both a centralized and decentralized manner. Over large geographical area a group of fog nodes can be monitored and managed in a centralized way. Cloud size is large as compared to fog, as it has massive storage of data from the Internet. Fog size is a flexible parameter that can be altered according to the demand of users. For example, for a vehicle tiny sized fog would be enough while for an institution many small fogs can work in a form of network or may be a large-sized fog would be sufficient. Fog has fewer deployment complexities as compared to the cloud. Similarly, cloud management is tricky and more time taking as compared to a fog because of its flexibility. This flexibility will support mobility in networks.

Because of architecture complexity, the cloud can only be operated by technical experts while fog can be managed and operated by little human effort. Large companies control cloud networks, while fog can be controlled by small as well as large companies. Low-latency critical IIoT applications will be handled by fog, while applications that can tolerate large delays leverage the cloud services. Internet connection between user IoT devices and a cloud server should be reliable for the entire time of connection, while in fog architecture connection with cloud is not necessarily required for the whole time. Bandwidth requirement increases for both frameworks as the number of IoT devices is increasing exponentially. It provides awareness property, which means it knows about the requirements of customers and will provide solutions accordingly. It can be placed anywhere between the cloud and user nodes according to the demands. Performance and efficiency parameters of IIoT application services can be enhanced using fog framework. In addition, the scalability issue can be handled using it. Data storage at network edge will result in minimization of service delays and supports real-time processing. The big amount of data produced from heterogeneous smart devices requires huge data storage and computation at the cloud server. IIoT applications requires data storage at their backbone. This big data is giving rise to edge computing for future. Edge computing is also called Mobile Edge Computing (MEC). This technology supports IIoT applications by building better operational connectivity. It brings cloud computing capabilities at the devices that are present on the edge of a network, these devices named as edge servers or edge devices. The edge devices are part of the Internet and participate in processing and computation near the data location. This technology is an industrial initiative by the European Telecommunication Standards Institute (ETSI) [41].

## 4. Protocols/Algorithms

Protocol is a basic set of rules that defines how communication happen between different devices in a network. Protocols have to be devised intelligently in order to achieve the defined goals. As the future is industrial automation in industry 4.0 revolutionized era, there is an exponential increase in smart, intelligent devices in IIoT applications. A major requirement is that the emerging network protocols must meet required goals in performance affecting parameters, such as energy efficiency, latency minimization, spectrum efficiency, cache memory maximization, and bandwidth use requirements. A summarized discussion on pre-existing protocols in the context of fog computing proposed by researchers is given below in following subsections.

### 4.1. Routing

Dong et al. [42] have introduced redundant fog loops for WSNs. The proposed fog loop-based scheme has two main steps. Creation of fogs using loop paths is the first step, while the second mechanism creates fog nodes in the source node areas along with many other interfering fogs within the network. This proposed scheme has helped in finding the exact location of the source node in terms of energy efficiency and privacy. Results were compared to the efficiency offered by the Phantom Routing Scheme (PRS). The proposed scheme gives improved efficiency by 4 folds and can also improve the privacy and security up to 8 folds.

Since fog computing lowers latency and offers energy saving, they are tailor-made for dealing with WSNs [43]. Sensors in WSNs are resource-constrained, therefore energy efficiency is an important issue. It needs to be addressed for the network to increase network life-time of operation and working efficiently for a prolonged period of time. Sensors in a form of clusters collected data and send to the base station using energy-efficient routing protocols. In this approach, using multi-hop communication, data is transferred to the sink/destination node. The nodes acting as a cluster head are used for multi-hop communications. For networking, apart from the routing problem, another issue that needs to be catered is increasing network lifetime. Network lifetime can be effectively increased by optimizing energy and power consumption at nodes. Some examples of these routing protocols are Low-Energy-Adaptive-Clustering-Hierarchy (LEACH) and Stable-Election-Protocol (SEP). LEACH protocol involves Deterministic Cluster Selection Head and abbreviated as LEACH-DCHS [44]. Handy et al. [45] have proposed LEACH in which rotation of the cluster heads in a randomized manner is used for energy distribution to the nodes evenly. These algorithms haven’t been used for fog computing yet, but can be used in future incorporating fog computing. SEP was proposed by Smaragdakis et al. [46], prolongs the period of stability of wireless sensor networks. A modified version of SEP (M-SEP) proposed by Singh et al. [47] is suitable for heterogeneous WSNs. Some of the nodes in this approach might have a greater chance of getting selected as a cluster head and have more energy. Moreover, in large WSNs, the data to be processed from heterogeneous devices is in large volumes. Processing time is significantly large, so an alternate approach is to use fog computing. The time required to process large amount of data gets considerably reduced using fog as the sensors in the network get interconnected with the Internet in order to make smart by making them autonomous in making decisions.

Another routing protocol that is energy-efficient named as new-SEP is proposed by Naranjo et al. [48], prolongs the stability of sensor networks supported by fog more than SEP. Optimal clustering nodes are elected by considering various sensor node features such as the ratio of heterogeneity in the network, residual energy, distance between cluster heads. Results were compared with baseline schemes of LEACH and SEP, the proposed scheme performs better in terms of energy-preservation and network lifetime. Considering increasing the network’s stability periods, N-SEP performs better than LEACH (50 percent) and SEP (25 percent). An efficient route optimization algorithm was presented in [49] to address the mobility control issue in fog-based SDN networks. The proposed SDN-enabled fog computing architecture had three-layered structure namely, fog layer, network layer and application layer. Results showed that there is a great improvement in network performance. A three-layered plane architecture was proposed to generate efficient routing paths by the authors in [50] for data-center-based heterogeneous networks, using tensor decomposition methods. These three planes do three different tasks, edge plane considers the traffic, bandwidth and delay requirements; fog planes computes and controls the available paths and finally cloud plane do the routing.

### 4.2. Resource Allocation

One way to meet the growing IIoT application requirements is to use fog computing. Interactions in real time takes place in fog application rather than batch processing. Services supported by fog include mobility, heterogeneity, working with cloud to extend cloud services, user optimization, etc. Resource allocation or resource management in any network is one of the biggest challenges. This defines new protocols for networking and communication. Many researchers have done work in this context and already proposed some protocols. Resource allocation was done with a specific defined objective, it can be maximizing energy efficiency or throughput; minimizing latency or power consumption or network cost. A joint min-max optimization problem of resource allocation and offloading decision making was proposed by the authors in [51]. They have proposed Computation-Offloading-Decision-Making and Resource-Allocation Algorithm (CORA) to minimize the maximal network cost (delay & energy consumption). Results were explained using fractional programming theory and lagrangian dual decomposition. Fog Radio Access Networks (F-RAN), an extension of the Services provided by Cloud Radio Access Network (C-RAN), has gathered attention globally and several advantages can be taken by providing functions of baseband signal processing near the edge or making the edge devices cache-enabled. F-RAN is the best example in which we are using edge devices as well as network devices (access points) as fog nodes to achieve the best results. Moreover, due to the cooperative communication, benefits of C-RAN such as enhanced spectral and energy efficiency are also conserved in F-RAN. Resource allocation is a challenge for the upcoming F-RAN. Authors in [52] have proposed a Stackelberg equilibrium (SE) for a hierarchical problem of network slicing customization between global radio resource manager (GRRM) and local radio resource managers (LRRMs). A game is formulated to alleviate the burdens in GRRM and LRRM; GRRM assigned resources to each slice and then LRRM in every slice provides resources to UEs. UEs could be divided into clusters on basis of respective objective functions and accordingly get resources. Authors have provide two algorithms for LRRM 1 slice and LRRM 2 slice with an objective function of maximizing high data rate and minimizing latency, respectively.

To meet low latency, high throughput and connectivity requirements in future RANs, non-orthogonal multiple access (NOMA) is a promising technique. Zhang et al. in [53] have formulated a problem for maximizing the net utility under interference constraint for resource allocation problem for NOMA-based FRAN system model. Results were compared with the conventional orthogonal frequency division modulation (OFDM) technique. Madsen and Albeanu [54] discuss platforms involving fog computing in which there is communication going on between the smart devices, fog, and cloud. A model for internet applications in future is presented by Hong et al. [55] in which applications are delay-sensitive and distributed geographically. For improving the rendering performance of a webpage, Zu et al. [56] exploit information available only at the network’s edge. Companies that want to deliver content such as Netflix uses fog computing to reach their geographically distributed customers. As indicated by [57], ensuring significantly large streamed data to be delivered in the proximity of the end-user (customer), is done using fog computing.

Minimizing the energy consumed in geographically distributed applications and resource allocation using fog computing is discussed in [58] for video services. Size of fog nodes deployment shows the application demand in that region. To maximize social welfare, some of the user data needs to be controlled. Optimization on large scale is also possible using proximal and distributed algorithms. The algorithm proposed gives a near-optimal solution.

### 4.3. Load Balancing

When one edge device has to do a lot of work e.g., computing tasks all by itself, it consumes resources. However, this can be reduced with the help of distributed architecture in which load is evenly distributed among the edge devices present in the network. This distribution of load is called load balancing. Load Balancing in combination with fog computing makes a formidable combination. A fog network can provide a platform for cooperation and coordination between edge devices in a load-balanced network. Authors have proposed a load balancing offloading algorithm for latency minimization in Vehicular fog computing (VFC). VFC is an integration of vehicular networks and fog computing technology, which is an efficient field to achieve real-time and location-aware for vehicles in a smart city. Zhaolong et al. in [59] has formulated a response time minimization problem using a three-layered decentralized network system model to balance the traffic among vehicles. These three layers are cloud layer with a high computing cloud data center, cloudlet layer processed the received data from vehicles before sending to the cloud center. Lower layer has vehicle clusters, clusters are made for traffic balancing. The fog-vehicle interface manages and alleviates the traffic load.

Traffic overhead results in inefficient resource management, which is also a challenge to be solved for healthcare applications. WSNs based health monitoring systems have become a convenient choice as elderly people can frequently require health services. The basic requirements for healthcare applications are energy efficiency, high-response time, low-latency and real-time connection. Fog computing can be considered to be an important enabling technology for such time-sensitive applications. Authors in [60] provide a very significant critical review of existing solutions provided by researchers in the healthcare domain using fog computing. The authors in [61] proposed a fog-cloud hybrid solution to load-balancing problem. If a client’s requirement is more critical, it will be handled by cloud otherwise servicing is done by foglets. Results shows that network utility can be enhanced in terms of latency and load balancing. They used iFogSim tool for experiments. Forough et al. [62] proposed an Energy Balancing Algorithm (EBA) for Fog-IoT networks to reduce delay and energy consumption. The authors have proposed two optimization problems, first one is to find an optimal transmission rate and power for terminal nodes (TNs) and the second one is to find an efficient topology between TNs and fog nodes (FNs). The system model was designed under the constraint of channel conditions between TN and corresponding FN. A major challenge in edge computing is the inefficient deployment of resources as it reduces the overall efficiency of the network. This results in definite increase in power consumption. A low service block is preferred for maintaining lower latency, which will otherwise be detrimental to the high-performance requirements of edge computing. Unlike cloud computing, fog computing has a limitation of resources, which can be overcome if we allow cooperation between various data centers. A cooperative scheme by Beraldi et al. [63] is proposed in which data centers near the edge exchange processing requests and shares the load of highly loaded data centers. The request arriving at a busy data center is forwarded to any other data center having the request buffer partially filled. The proposed scheme maintains a threshold of requests with the help of Markov Chains to make sure that load is equally distributed among all the data centers. This leads to much-improved performance as lightly loaded data centers can absorb the burden of heavily loaded ones during peak hours.

Full use of edge resources cannot be done by cloud computing polymerization calculation [64]. The edge devices are not part of the cloud computing, which is undesirable for delay-sensitive requirements. Ningning et al. [65] have explored how fog computing can turn the nodes or edges into virtual machines using Cloud Atomization Technology to improve on this problem. The authors use graph partitioning for developing an efficient load balancing algorithm. Consequently, a flexible network can be built by making fog networking possible after atomizing the cloud and eventually reduce the cost of the system that were high before implementing the load balancing algorithm. Deng et al. [66] investigate power consumption and transmission delay trade-off. Problem of workload allocation is formulated to obtain the minimal power consumption subject to the constraint of service delay. Three sub-problems result after the decomposition of the primal problem. Based on the obtained results, it is shown that cloud performance is enhanced by using fog computing. Moreover, latency is reduced and bandwidth can be saved by sacrificing some of the computational resources. Computational clusters are formed on the basis of cooperation behavior between the Small Cells (SC) to share computational resources. However, the cooperation is dependent upon many factors such as resource availability, resource allocation, delay constraints of the application, distribution of computational load and size of the cluster. The joint distribution of resources for the mobile end-users and the cloud is the main objective problem of this framework.

All the data that is being frequently accessed by the edge devices is stored at the Radio Units (RU), which considerably decreases the overall delay in the network of Fog-Radio Access Networks (F-RAN). However, in such systems, the energy efficiency aspect has always been a matter of serious concern due to the addition of extra smart components in the system. In [67], the authors have proposed a novel scheme by designing a green network in which an efficient algorithm is incorporated to optimize the selection of RU. Furthermore, the algorithm also jointly optimizes the formation of clustering and beamforming while maintaining the QoS and balancing the load of each of active backhaul as a measure of its capacity. While copying data from the database, edge devices may interfere with each other. To avoid this mishap, data replication techniques are used for copying data electronically from the main database where the data of all the users is hosted. Uniform distribution of data and processing is crucial over the network which in turn helps in managing the large amount of data and workload with efficiency. Fog computing not only helps in achieving higher efficiency, it also helps in balancing load across distributed platforms and achieving higher energy efficiency due to fewer performance bottlenecks. Verma et al. [68] have focused on making a network that is less dependent on cloud computing and bringing the storage and processing capabilities near the edge devices. The results are simulated with the help of CloudSim by testing different geographically separated servers and their configurations and then make comparisons between cloud and fog computing for various attributes.

## 5. Challenges with Solutions

In general, there are many challenges towards industry 4.0 digital transformation. For M2M communication, reliable and stable connectivity with bounded delays is a mandatory requirement. Real-time communication is on the higher priority for this fourth revolution, which brings many technical challenges for future network development. A well-designed network architecture can increase the sustainability and performance of the entire system. The journey towards industry 4.0 is on its way and until now there are no such standards, regulations, and certification to follow them.Although fog becoming a developing key enabling technology for IoT architecture, it still faces some issues while integrating into the current architecture. Complex software applications and solutions are needed to achieve an efficient fog network. Fog network will be analyzed in terms of key performance measurements, which are bandwidth use, energy consumption, low latency, maximum throughput, and resource management. In this section, critical technical communication and networking challenges in the context of fog computing for IIoT applications are listed. All of them have potential for future work.

- **Power Consumption/Energy Efficiency:** Several smart devices supporting an IIoT application will consume a massive amount of energy on a different scale according to their requirements. Ensuring network QoS with minimum energy consumption of smart IoT devices, fog nodes and cloud in an optimized way is an open challenge for every upcoming future IIoT application.
- **Throughput/Rate/Capacity:** Throughput or network bandwidth, data rate and storage capacity depends on how much data is used and where data is stored in a fog network. This data placement on fog nodes or edge devices or cloud server has effects on cost, delays, bandwidth, and network coverage. The optimal placement of data on cloud server or fog cloudlet is one of the critical technical challenges for fog-IoT architecture.
- **Spectrum Use/Resource Allocation:** Geographically separated fog, cloud nodes and their interconnection makes the backbone of any network that relies on offloading services. Most of the cloud computing interconnection mechanisms are not enough for fog networking due to their limitations including relying on a centralized cloud which cannot fulfill the latency and location awareness requirements of distributed devices, etc. Fog computing must encompass features, such as multi-tenancy, scalability, heterogeneity and quick resource provisioning. An architecture including fog and cloud computing must meet all these requirements for which resource allocation/use is the most critical challenge for better network performance. It has effects on all other QoS parameters.
- **Latency:** IIoT applications requirement is real-time connectivity. All applications are time-sensitive and require real-time streaming rather than batch processing. Fog computing gives a better result for such decentralized solutions. It gives low latency with reliable connectivity and mobility. Optimized placement of data centers, resource allocation, network architecture, energy consumption of nodes, and storage capacity of nodes have impact on latency. Latency for a network is the sum of transmission, processing, propagation and queuing delays. To achieve the low-latency requirement, there is need mitigate all types of delays.
- **Cache Enabled Edge Devices:** Caching content locally, reduces the access delay time and increases the energy and spectral usage efficiency. Since the Internet has multiple bottlenecks while accessing data from across continents and oceans, caching does not have to be dependent on any of these bottlenecks and instead makes the same data available locally. Furthermore, caching incredibly reduces the load on backhaul links since they do not have to be used anymore for accessing data. Since all the users have to access the data from the same centralized location (internet/cloud server), a certain degree of fairness is needed to avoid inefficiency in accessing data. Since all the backhauls have certain capacity constraints, there is a need for an efficient load balancing mechanism to overcome this issue.

Further, we discuss existing solutions to these mentioned challenges.

### 5.1. Power Consumption/Energy Efficiency

Objects are extensively being connected together using the IoT technologies. Heterogeneous smart objects, in the context of hardware and software, can perform efficiently in the availability of memory and high computational power. Network’s complexity increases day by day, because of scalability issues of smart objects supporting IIoT applications.

Virtualization has played a very crucial role in improving the overall efficiency of the data centers and now research has also been done on how virtualization can help in fog networks. Virtualization makes it easiest to deploy fog functionalities on an existing node (by isolating and securing fog services in a virtual machine or container). It also helps in conserving energy by efficiently consolidating the tasks on a single fog node. A comparison in terms of power consumption was studied in [69] between C-RAN and F-RAN using network function virtualization (NFV) technology. The authors have formulated a mixed integer linear programming (MILP) problem, results for F-RAN are 30% more improved in terms of power-saving as compared to C-RAN.

Roca et al. [70] have developed a platform using Fog-Function Virtualization, which works and builds on the concept of Network Function Virtualization (NFV) for multiple IoT applications. This, further with the help of node constellation creation, helps in easy deployment and reduces the cost considerably as less energy is required for running the system due to efficiently performing virtualization. Task scheduling is necessary as it helps with the load balancing aspect of networking and can provide services to multiple users. Cooperative games between the containers and brokers are studied for energy-efficient task selection algorithm. Kaur et al. [71] have achieved efficiency with the help of container-as-a-service (CaaS). Lightweight containers have been used which considerably reduce the energy consumption by a container migration techniques. This kind of virtualization is more cost-efficient for distributed architecture, where a large number of devices with different running applications/processes can be allocated to resources efficiently. Results achieved by the authors prove that the system is more energy-efficient.

Graph-Based Heuristic algorithms were proposed by the authors in [72]. Type of problem is the integer linear programming (ILP) problem, the objective is to increase the energy efficiency under association and capacity threshold constraints for hybrid cloud-fog RAN (CF-RAN) architecture. Minimization of latency and power consumption is also addressed in the proposed system model. Fog computing along with NFV gives excellent outcomes in terms of reducing latency and power consumption. Energy can be saved by incorporating techniques, such as Message Queue Telemetry Transport (MQTT) in a fog-based environment [22]. In this scheme, the number of transmissions is reduced to save energy of the end devices. Using energy-efficient routing protocols is imperative to achieve energy efficiency. MQTT supports sensor data in real time due to its many-to-many communication nature. The concept of MQTT focuses on introducing another layer between the fog and cloud with lower complexity. MQTT broker place is at the fog layer. The intermediate layer is responsible for predicting the future measurements, and acts as a gateway for the upper layer. It helps to offload the computationally expensive tasks from the cloud to save in the storage memory of the fog server. This results in a reduced number of transmissions as the update only occurs in case of a mismatch.

### 5.2. Throughput/Rate/Capacity

For network designing, a new paradigm known as Socially-Aware-Networking (SAN) has been of major interest [73]. To achieve efficient performance, SAN brings the human behavior and CPS together via intelligently designing of a network. This design should be adaptable as well for all environments. The resources available to mobile devices differ depending on the models and specifications. This resource availability results in a group of mobile devices that might be sufficient in terms of processing and storage parameters. Group of some might not be self-sufficient. The best solution to this problem is given by SAN and Fog-Radio Access Networking (F-RAN).

D2D communication comprises the direct sharing of contents among mobile devices. Direct sharing is a key feature supporting D2D communication. For achieving efficient performance results, an imperative design of network embedded with all technologies is required. Research has shown that the system performance in terms of utility, throughput and energy efficiency is maximized using the download mode with the help of branch and bound algorithm [74]. Klas et al. [75] and Cau et al. [76] worked on improving network efficiency Mobile Edge Computing (MEC). MEC is a domain under fog and focuses on providing cloud computing capabilities near the edge of network. Efforts and improvements have been made in MEC in order for it to support 5G communications. Another target of MEC is to achieve access to information provided by the radio network for application development and distribution of the content. It is predicted that data traffic over mobile devices will increase manifold in the next few years. Such increasing needs must be satisfied using efficient mechanisms.

A reliable service with an extremely low latency (URLLC) and high capacity is the foremost requirement of IIoT networks. Throughput maximization of the F-RAN system is compared with three different back-haul strategies for Small Cell Networks (SCN) in [77]. All these strategies namely, decode and forward, direct transmission and C-RAN were studied under delay threshold, rate constraint, and backhaul and fronthaul links. The authors proposed iterative algorithms for all strategies. For F-RAN, Pontois et al. in [78] formulated a non-convex optimization problem under fronthaul constraints. A hybrid semi-distributed resource allocation algorithm was proposed by the authors for the proposed weighted sum-rate maximization problem. Results show that there is a trade-off between maximum throughput and system latency. A multi-objective optimization problem was proposed by the authors in [79]. They have proposed three parallel algorithms to improve latency, throughput and resource management. A queuing model was studied under task buffering, offloading and resource allocation algorithms. Lyapunov drift was used by the authors to design the resource allocation policy. In results for better system performance, trade-off between latency and throughput is observed. [80] end-to-end performance is guaranteed after composition of problem as Multi-Constrained Optimal Path (MCOP). The authors propose a solution from the network architecture’s perspective and cloud service relation. The proposed algorithm provides better results in terms of efficiency and effectiveness. QoS parameters (capacity, delay and cost) gets improved by the proposed network-cloud service provisioning system model.

### 5.3. Spectrum Use/Resource Allocation

Fog networking can provide a solution for resource management issue in future 5G networks. With a growing number of smart devices, the major issue is of spectrum use and resource allocation. Spectrum pricing and allocation scheme (SPAS) was proposed in [81] for F-RAN framework. Three areas were defined for the whole network, and double game theory was applied to give the solution for efficient spectrum use. Proposed two algorithms are named as game-model (GM-SPAS) and multiple-spectrum-reuse-technologies (MSRT-SPAS). Their presented results are effective in terms of revenue and efficiency. The problem of resource allocation with utility maximization objective under QoS constraints was formulated in [82]. Analytic Hierarchy Process (AHP) was proposed by the authors for finding the QoS requirements of IoT devices. Afterward, the matching theory was applied for user association.

In [83], the authors have used the calculus of variation to find out the optimal spatial density of nodes such that the distribution of nodes can support the whole network. A Parallel Imperialistic Competitive Algorithm (PICA) is used to determine the initial positions of the access points. WSNs with immense scalability, immobility factor and low-deployment cost properties have limited processing and are resource-constrained. Smart Mobile Access Point (SMAP), which has been proposed by Majd et al. [84] aims at achieving higher resource use and energy efficiency with the adoption of hierarchical placement of SMAP in WSNs using fog computing. Moreno-Vozmediano et al. [85] have presented a framework for interconnection of fog and cloud computing, Hybrid Fog and Cloud (HFC), that provides effective, simple and productive resource provisioning. It also automates the process of configuring various virtual networks that are part of the network for interconnecting important components. The proposed architecture covers the salient features, such as security and scalability along with fog to fog, and fog to cloud communication mechanism.

Shojafar et al. [86] propose a resource scheduler for Networked Fog Centers (NetFCs) that not only provides services to the vehicular client but is also energy efficient. NetFCs operate from vehicular network’s edge via Infrastructure to Vehicle (I2V) mobile links to the vehicular clients that are being served. Not only does the overall computation and communication energy efficiency get maximized but also the network performs better in terms of QoS requirements. Moreover, hard QoS requirements induced by the application are met, such as reducing transmission rates, delay, and jitter. Resource scheduler is responsible for admission control, dispatching the allowed traffic using minimum energy and adaptively controlling the traffic that is being injected into the mobile connections. Few of the important characteristics of the scheduler include providing QoS guarantees induced by the application, implantation of the scheduler is both scalable and distributive.

### 5.4. Latency

Fog networking is a key paradigm to provide solutions to latency-sensitive future IIoT applications. Many researchers have contributed to cater latency minimization problem. Online Fog Network Formation Algorithm was proposed by Gilsoo et al. in [87] for minimizing the overall maximum latency (communication and processing) of a fog network. The objective problem constitutes of the sum of two types of delays i.e., fog network formation and task distribution. A joint energy and latency optimization (JELO) scheme for F-RAN was proposed in [88]. The joint optimization of energy consumption and latency was formulated as an integer-programming (IP) problem subject to user association, capacity and latency threshold. The proposed complex problem was divided into two sub-problems of knapsack and semi-assignment problem. The proposed algorithm gives better results as compared to the existing techniques in the literature. A latency minimization problem was formulated for IoT-fog network under user association, workload and latency threshold constraints. The authors have proposed a matching-game theory for the proposed resource management problem for network latency minimization [89].

Over the last few years, the Internet of Vehicles (IoV) has been a matter of growing interest. Cloud computing provides high performing IoT services to IoV, still, there are many shortcomings when it comes to mobility support, latency and location awareness. Xiuli Hi et al. [90] have integrated fog computing into SDN. While fog Computing helps with the latency of the network, SDN provides flexibility in the centralized control and providing the complete global knowledge of the network. Vehicle to Vehicle (V2V), Vehicle to Infrastructure (V2I) and Vehicle to Base station (V2B) communication can be supported by a weighted undirected graph having Road Side Units (RSUs). The simulation results given by the aforementioned authors decrease the latency and achieve a higher QoS. The scheme proposed by Skarlat et al. [91] tackles an optimization problem, providing a solution that there is no trade-off between communication and using resources and energy for computation. The applied system model demonstrates that in optimization scenarios, reduced average round-trip time (RTT) and delays up to 39 percent can be achieved using fog. The scheme also considers an independent cooperative number of working nodes.

For the smart city, Fairness Cooperation Algorithm (FCA) was proposed by Dong et al. [92], for the joint optimization problem. The authors have formulated a convex-non-linear programming problem of minimizing the total system cost (delay and energy consumption), subject to power, workload and computation capacity threshold constraints. QoE and fairness of users under FCA was compared with baseline algorithm (BA) and distributed optimization algorithm (DOA).

### 5.5. Cache Enabled Edge Devices

Fog networking is a vast field, which uses both smart objects and the already deployed network infrastructure. System can ensure QoS either by using already deployed network devices or by optimally dividing the tasks among edge devices or using both at the same time. Cache placement in edge devices/fog nodes in a network improves the efficiency of a system in terms of low power consumption, low-latency, high throughput, and efficient spectrum use. To deal with the congestion problem at the backhaul link of F-RAN, authors have proposed a projection gradient method in [93]. To increase the throughput, they have used stochastic geometry to derive a close-form of successful transmission probability (STP). Afterward, the optimal placement of cache over the network was done. To fulfill the delay requirements, authors in [94] have proposed a decentralized asynchronous coding caching scheme. The result shows that the proposed algorithm is more efficient than existing algorithms present in the literature. A convex optimization problem of minimizing the worst-case fronthaul delay was formulated by the authors. Depending on application delay requirements, the proposed coded scheme gives synchronous and asynchronous transmission methods. The problem of user association with a fog node on the basis of lower latency is formulated in [95]. The authors have formulated the problem using game theory, for which they have used a proactive caching scheme and Boltzmann-Gibbs learning algorithm for solution. Latency of proposed fog network is the sum of computing and queuing delays.

Different researchers have worked on the F-RAN design considering various aspects. For a green system, as discussed by Chen et al. [67,96], minimization of energy consumption is done by considering an F-RAN that is cache-enabled for the selection of Remote Radio Head (RRHs). For balancing front-haul traffic, Park et al. [97] have discussed a scheme to deliver data from Base Band Unit (BBU) and RRHs. Content placement problem involving caching has been discussed in X Peng et al. [98] and Dai et al. [99]. F-RANs accommodate caching in the road-side units. Di Chen et al. [100] have worked on maximizing the Signal to Interference Noise Ratio (SINR) to ensure fairness while jointly optimizing cluster formation and multicast beamforming. The objective function in this work has non-convex constraints and is collectively an NP-hard problem. Sensor-cloud system gives solutions to many applications in a smart city. The system was developed by the integration of CPS and cloud computing. Besides many benefits, a major problem is coupling resource management, which was discussed in [101]. They have introduced a fog layer between sensor and cloud layer, which emphasizes the services. Firstly, authors have proposed an algorithm for caching at fog layer, afterwards, Hungarian algorithm was extended to deal with the optimal use of resources on basis of maximum matching. The proposed algorithms result in the minimization of latency for sustainable services.

## 6. Open Research IIoT Application Domains, Fog Computing as an Enabler

In Industry 4.0, the digital transformation of the industry requires research development in all fields (smart city, D2D communication, transportation, healthcare, etc.). These all IIoT domains have same critical issues in communication and networking. Even though, fog computing has umpteenth applications in multiple research areas, few notable applications in respective of IIoT domain have been listed in Table 1 to gain an idea about the diverse use of fog computing. The main objective is achieving maximum benefits/solutions using fog computing for this industrial revolution at efficient optimized QoS measurements. Many authors have proposed solutions in past years to networking and communication challenges in order to leverage benefits using fog computing in all domains of IIoT. Some has started to provide prototypes for supporting their research, Table 2 has listed some case studies for various areas of open research. In this section, some of the past research works are summarized for readers to get an idea about the integration of fog computing with different pre-existing network architectures. This integration brings solutions to support IIoT applications, yet there are many open research areas in all fields that need to be solved in the coming future towards the industrial development.

### 6.1. Micro-Grids (MGs)

Multiple loads and distributed renewable energy resources combine to form an electrical system named as MG. This energy is stored in the storage devices, though, it can have significant power losses when power is exchanged between different MGs. It increases reliability and efficiency of the system. In [104] authors implemented their proposed framework on a test MG system. Performance was observed for the proposed three-layer fog computing system. A convex linear programming problem was formulated with an objective of minimizing the total cost in terms of power consumption. The constraints are total load threshold which was calculated using the power balance equation. The equation has three types of powers with threshold range namely, dispatchable, non-dispatchable and grid power. Graph theory was applied to the proposed system and a fast consensus-based algorithm by taking advantage of fog computing was proposed. An optimization problem of power demand problem was discussed in [105] for hybrid fog-cloud system. The high volume of data by the number of smart devices results computation delays at cloud also it involves more power consumption. This power consumption can be optimized after load balancing among fog nodes and cloud server. Jalali et al. [134] have discussed various ways for the deployment of IoT nodes in an energy-efficient way. The authors have used MGs and fog computing as an enabler with an aim to reduce the energy consumption by the IoT specific applications. Energy consumption of various types has been discussed i.e., energy consumed during computation and balancing traffic on fog as well as the cloud. Renewable energy is stored in MGs, which can be used for further processing. Dynamic decisions such as weather forecasting or renewable energy availability causes dependent energy-saving processes.

### 6.2. Smart-Grids (SGs)

Due to an increase in the number of devices required by a user at home, residential usage of electricity has been increasing with time. The proposed modern solutions, such as SGs, greatly improve the reliability, efficiency, and sustainability of the system in an automated way. Using communication technologies, SGs works on gathered information about consumer’s and supplier’s behavior. As SGs are distributed systems implemented on a very large scale, fog computing is ideal to deal with such a scenario. For this purpose, Foteini Beligianni et al. [135] have discussed how IoTs and fog computing can be integrated into the power systems without compromising user privacy. The proposed architecture works on demand-response service to ensure privacy by using standard protocols and open-source resources. For devising a power plan for all the devices, a load scheduler is placed in the system while data management performs all the necessary actions that are required to process the data. The software architecture of the proposed solution is based on Lambda architecture which embodies edge computing. To meet the demands of real time processing, collecting information, computing and storing the data generated by multiple smart meters, the aforementioned researchers have proposed a fog-based solution. Fog networking acts as a bridge between the cloud and the SG. With fog networking, geographically distributed smart meters can be employed. Latency is reduced while improving location awareness and privacy for SG. Furthermore, Rao et al. [136] have worked on how to maintain the QoS by minimizing the expense of electricity using coordination between data centers.

### 6.3. Multimedia

Multimedia communication involves a large amount of audio and video data, being produced by smart IIoT devices. This ever-increasing amount of multimedia content brings new challenges. Low-latency and energy-efficient solutions are required to provide services. A direct consequence of increasing multimedia traffic is overburdening of the already strained mobile access network channels. The essential components of a multimedia communication system can be visualized with the help of Figure 1. The authors in [106], have formulated a cost minimization optimization problem, this cost depends on scheduler decisions. Scheduler takes decisions regarding distribution and states (open or close) of fog nodes. This cost minimization problem alternatively converted into multimedia user’s (MMU) response time minimization problem under capacity, coverage zone area and association constraints. The authors have introduced a fog node to resolve the resource management and latency issues between cloud and MMUs. Using Stackelberg game, an online resource allocation scheme was proposed. The enormous amount of data production for smart applications, need security and privacy as well. The authors have proposed a chaotic cryptographic method to ensure security along with low-latency requirements for Information-Centric Multimedia Network (ICMN) [107]. The speed of encryption and decryption of multimedia streams in the cryptographic method, was enhanced using fog computing.

### 6.4. Device to Device (D2D) Communication

Researchers have to find a new way in which devices can independently work without using the existing cellular infrastructure. The demand for new infrastructure development is due to the increase in the number of smart devices that causes scarcity in spectrum and resources. This idea gives rise to D2D communication technology in which devices are part of the Internet and can communicate with each other without the involvement of internet infrastructure. This direct communication will result in less usage of the wireless spectrum. A device is itself constrained-bounded in terms of parameters, such as energy or resources, hence cloud and fog computing technologies support them. These emerging technologies enhance processing capabilities and result in efficient system formation. Cloud services for D2D communications have performance bottleneck as devices use direct communication, hence an alternative fog service is required. Fog with the same set of services, provide better performance. Fog networking act as an alternative of cloud, as it provides desired requirements in the vicinity of edge devices. Researchers have proposed D2D fogging to address energy-efficient task offloading in D2D communication [137]. The proposed method includes a framework for task offloading and uses assistance from the network for D2D. Mobile users use the communication and computation resources of each other dynamically. After discussing several security issues in fog computing, authors have proposed three lightweight anonymous authentication protocols (LAAPs) [108].

The proposed scheme with the aid of D2D communication, is feasible for IoT devices which are resource-limited. Li et al. [109] proposed a F-community architecture for F-RAN followed by a data caching scheme. The caching scheme for UEs in D2D aided F-RAN helps in reduction of delays. Nodes in the system with higher chances of being selected as the central nodes store the most popular content in their cache. For access to any data, a user receives data from the cache stored previously. Kaur et al. [138] have proposed cachinMobile, which, compared to the cloud, can meet the low latency and energy efficiency requirements by using the elastic services provided by the nodes at the edge. Energy efficiency and low latency requirements is a major issue in this technique as the resources at the edge and the devices that are mobile. This approach has proposed to use D2D communication to carry out communication at a short distance and save network resources. CachinMobile not only improves energy efficiency but also maintains QoS.

### 6.5. Vehicular Ad-hoc Networks (VANETs)

This area is named as Intelligent Transportation System (ITS), which is an emerging area with many open issues that need to be solved. A transportation service and automobile service management involves the controlling and monitoring of the transportation network. ITS is designed in such a way that this system can satisfy the required QoS parameters of the transportation network. These QoS requirements involve reliable connection, efficient performance, safety, and privacy requirement, mobility and scalability requirement. A transportation network system aided with ITS technology has components that can be optimized. These are Global Positing System (GPS), RFID sensor tags and readers, road-side equipment for example traffic lights, signals, road bank cameras, cars. These ITS subsystems integrated with IoT technology elements will help in monitoring and managing of transportation environment; distribution of vehicles according to the scenario; manufacturing of ITS; shipping, tracking and monitoring of physical objects.

Enormous number of such physical objects embedded with data processing capabilities, integrated with RFID sensors along with networking technologies will promote IIoT applications. These IIoT applications will help in monitoring the exact original location as well as destination location of vehicle or aero-plane or ships along with the followed path traffic condition or environment effects or any emergency road situation. Many authors have done research in designing ITS supportive transportation networks optimizing wireless communication technologies, RFID tags or antennas. The use of IoT technology in the transportation industry and systems results in new research domains such as vehicular ad-hoc networks (VANETs), internet of vehicles (IoV) and vehicle to grid (V2G). These domains are very promising areas of research. In recent times the integration of VANETs with fog computing results in better efficient system designing. Services of automotive connectivity architecture can be improved using fog computing as an enabler for smart transportation (ITS), such as the one shown in Figure 1 (Transportation). Efficiency is improved in terms of minimizing the latency for time-sensitive applications using fog computing. Being an integral part of the ITS, VANETs have many applications. Computation and communication demands are hard to meet in the cloud architecture. Incorporating fog networking not only fulfills the demands but also improves latency, location awareness and energy efficiency. For power management, there is a need for new technologies. For this problem Vehicle to Grid (V2G) is a recent concept with open issues, that uses renewable energy. When renewable energy is available it is used to charge plug-in electric vehicles. Otherwise, these electric vehicles are used as the source of energy. The use of alternative energy source will reduce the power burden on the grid during peak hours. Owners of these plug-in electric vehicles got paid by electric companies using metering systems. It provides some relief to electric companies in terms of payment, traffic load, and energy consumption during peak hours. In smart grids, a distributed architecture with storage and processing capabilities is need to be deployed, as there is a high factor of mobility in V2G. To implement the V2G services in the 5G network, a hybrid fog and cloud architecture was proposed by researchers in [110]. Open issues, such as energy efficiency, resource management, security, and privacy are needed to be addressed in the future to improve system efficiency. Security is a big challenge in VANETS, fog computing integration is smart transportation brings solution to this challenge. Ma et al. [111] have designed a new authenticated key agreement (AKA) protocol for fog-based VANETs.

The authors in [112] compared fog and cloud computing performance in a real VANET environment. Results shows that fog computing gives better services for real-time scenario applications, namely traffic detection and time estimation. Fog computing performs well because it supports the main attributes of VANETs that are location-awareness, mobility and real-time communication. Integration of the Internet of Things with VANETs gives rise to Internet of Vehicles (IoV) is a matter of growing interest over the last few years [139].

### 6.6. Big-Data Analytics

As the next revolutionized era comprises enormous smart devices that supports IIoT applications, this causes the generation of a significant amount of data. Due to the widespread acceptance of fog computing, significant research has been carried out in the domain of big-data analytics. Recent advancements show an indication that fog networking has the potential to provide solutions in this field. Fog computing, as an extension of cloud computing, gives solutions to problems such as location-awareness, mobility, big-data analytics, and cyber threats. Author in [119] have designed a three-layer architecture of IoT-Fog-Cloud that supports big-data analytics and security applications. Author summarized cyber attack types and compared the existing security solutions. The authors in [140] provide a review article on fog computing challenges in the context of big IoT analytics.

### 6.7. Software Defined Networking (SDN)

SDN is an evolving concept to remotely control the entire network from a centralized location. Distributed networks can be integrated with SDN architecture for more granularity of control in the network. Since edge computing is done near the edge devices, there is a high probability that the edge devices are mobile due to the exponential evolution of smartphones and other handheld devices. Similarly, the ever-changing requirements of resources for each user brings a certain degree of dynamism in the network which needs to be catered to ensure efficient operation. Since fog is a new concept and it cannot completely replace the existing cloud architecture, both fog and cloud work hand in hand for smooth operation of the network. The interplay between fog networking and cloud computing is always there when both work in an integrated fashion. The four-layer architecture was studied for end-to-end delay, energy consumption and packet loss ratio in [115]. The authors formulate mixed integer programming (MIP) problem of minimizing the energy consumption under data rate, bandwidth and delay threshold constraints. A new routing protocol was proposed for VANET applications using SDN and fog computing, named as Energy Efficient Multicast routing protocol (EEMSFV). SDN controller, OpenFlow switches and fog computing works under two algorithms, priority based scheduling algorithm for classifying the traffic message type (emergency or safety applications) and a classification algorithm to schedule the requests.

## 7. Conclusions

Industry 4.0, a revolutionized era which will have a massive number of smart devices that will support IIoT applications in every field. This deployment of smart devices will change all domains of human life’s perspective. IIoT applications will provide solutions to all fields, such as transportation, healthcare, food supply chain, education, and industry. These IIoT applications will provide efficient, effective solutions for future networks. There are challenges in communication and networking in terms of latency, bandwidth, resource allocation, and storage.

All these advanced IIoT applications will create a huge amount of data, causing a burden on the cloud. Even though cloud computing provides services to the edge devices, it incurs huge latency, resource allocation challenges, caching placement problems, energy consumption. These issues are detrimental to the QoS aspect of a network. Fog as an extension of cloud provides a platform to compute, control, store and manage these IIoT devices. In the future, it will reshape all sectors involving IIoT applications, with the integration of important existing communication technologies namely, CPS, SDN, NFV, 5G, D2D. This brings computation, resource management, and storage challenges. In this paper, first, we gave an overview of IIoT applications and its enabling technologies used for new revolutionized era. The pre existing protocols and solutions to challenges related to fog computing are summarized. In the end, we have mentioned open research IIoT domains, in which fog computing can act as an enabler. We have previewed the work carried out by numerous researchers incorporating fog computing to provide services to IIoT edge devices leveraging towards Industry 4.0 way. Critical review of some existing work is summarized in the table, which can be used to find open research challenges. Towards the development of this industrial transformative epoch, most of the research work is still uncertain and waiting. This is an interesting era to discover what fog computing may contribute to the world of automation in the coming future.

## Figures and Tables

**Table 1:** Literature Review: R.A=Resource Allocation, L=Latency, E=Energy, T/R/C=Throughput/Rate/Capacity, Cc=Cache, P=Power, H=Handover, B=Bandwidth, S=Security, T.L=Transmission Link.

Ref. No	IIoT Application Domain	R.A	L	E	T/R/C	Cc	P	H	B	S	T.L	Architecture
[49]	Mobility							✓			Routing	SDN
[50]	Big Data Analytics	✓	✓								Routing	HetNets
[51]	Smart IoT devices	✓	✓	✓							Downlink	Cloud Computing
[52]	Smart IoT devices	✓					✓				Downlink	RANs
[53]	Big Data Analytics	✓					✓				Downlink	NOMA+RANs
[59]	VANETS		✓								Downlink	Cloud Computing
[61]	Healthcare	✓	✓						✓		Downlink+Uplink	Cloud Computing
[62]	Smart IoT devices		✓	✓			✓				Downlink	Fog-IoT
[69]	5G network		✓	✓			✓				Downlink	NFV+RANs
[72]	Virtualized Passive Optical Networks (VPON)/5G		✓				✓				Downlink+Uplink	RANs + Cloud Computing
[77]	Small Cell Networks (SCNs)/5G	✓			✓						Uplink	RANs
[78]	5G network	✓			✓		✓				Downlink	RANs
[79]		✓	✓		✓						Downlink	Cloud Computing
[81]	5G network	✓							✓		Downlink	RANs
[82]	Heterogeneous IoT applications	✓					✓				Downlink	HetNets
[102]	VANETs	✓	✓									VFC
[84]	Smart monitoring systems	✓									Downlink	WSN+CPS
[85]	Security	✓			✓						Routing	VN+Cloud Computing
[87]	Heterogeneous IoT applications		✓				✓				Downlink	Cloud Computing
[88]	Time-sensitive IoT applications		✓	✓							Uplink	RANs
[89]	Heterogeneous IoT applications	✓	✓				✓				Downlink	Cloud Computing
[93]	Wireless network				✓	✓	✓					RANs
[94]			✓			✓					Downlink	RANs
[95]	5G network		✓			✓					Downlink	Fog-IoT
[103]	Microgrid	✓	✓				✓				Downlink	VM+Cloud Computing
[104]	Security+Microgrids						✓				Downlink	Cloud Computing
[105]	Microgrid		✓	✓			✓				Downlink	Cloud Computing
[101]	Smart city	✓				✓					Routing	CPS+Cloud Computing
[92]	Smart city		✓	✓							Downlink	Fog-IoT
[106]	Multimedia	✓	✓				✓				Downlink	Cloud Computing
[107]	Secure and time saving multimedia		✓							✓	Routing	ICN
[108]	Secure IoT applications									✓	Downlink	D2D
[109]						✓	✓				Downlink	D2D+RANs
[110]	5G mobile network+V2G services		✓								Routing	V2G
[111]	VANETs		✓							✓		IoT+ITS
[112]	Mobility+VANETs		✓								Downlink	Cloud Computing
[113]	Mobility+VANETs	✓	✓	✓			✓				Downlink	Fog-Ues
[114]	Mobility+Smart city		✓			✓						RANs+Cloud Comptinig
[115]	VANETs			✓					✓		Routing	SDN
[116]	Heterogeneous IoT applications			✓						✓	Routing	SDN+Blockchain
[117]	e-Healthcare									✓	Downlink	Blockchain
[118]	Cooperative+secure healthcare									✓	Routing	Fog+IoT
[119]	Big-Data Analytics+ security									✓		Cloud Computing
[120]	Smart home										a case study	Cloud Computing
[121]	Smart city video applications	✓							✓		Routing	Cloud Computing

**Table 2:** Case studies, Fog Computing as an enabler.

Ref. No	IIoT Application Domain	Case Study: Key Focus
[122]	smart city	Smart city solutions have been deployed in cities, such as Barcelona and Venice, to make further advancements in e-governance
[123]	smart traffic control and health monitoring	To increase flexibility in a fog computing in the context of Complex event processing (CEP), a case study is presented. The methodology, called “mechanism transitions”, is used to study how and where a query should be processed and how this decision affects the performance.
[124]	smart city	Fog Computing Architecture Network (FOCAN) is presented to give low-latency and energy-efficient solution for smart city applications. It manages different application’s requirements by categorizing the traffic type and its flow.
[125]	city, factory, building, home	Using an open-source platform Distributed Node-RED (DNR), authors have presented how applications can be decomposed and deployed. They build prototype for scalability and dynamic nature solutions using the network simulator Omnet++.
[126]	smart pipeline monitoring	A sequential machine learning algorithm on every layer of fog-cloud architecture, sensors and Markov model are used to monitor, control and detection of hazardous events of a pipeline system. A working prototype was constructed to observe 12 distinct events. This prototype could be used for future city-wide pipeline safety measurements
[127]	smart transportation	The extended policy management to support secure travel to user’s is presented by the authors. Four different route guiding scenarios are explained; namely depending on traffic condition, emergency connected vehicles (ECV), connected vehicle (CV) and probable collision detection.
[128]	smart transportation	Smart transportation framework is proposed for Vehicle to Vehicle (V2V) communication by the authors, on basis of the current traffic situation (road and vehicle’s condition, capacity).
[129]	big data	Case study named as “Streamcloud” is presented to provide real-time energy-efficient solution.
[130]	healthcare	Personalized missing data resilient decision-making approach is validated on a real human subject trial on maternity health. Data missing in critical applications is a very crucial challenge, that needs to be solved.
[131]	healthcare	Table 2 in the mentioned paper gives some projects for healthcare monitoring supported by fog computing, cloud computing, and IoT.
[132]	healthcare	A demo test-bed is developed on edge-IoT architecture for e-healthcare applications. Proposed EH-IoT gives better results towards bandwidth and latency requirements. The article also presents the benefits leveraging from IoT and edge computing from an industrial perspective.
[133]	cardiac diseases	A case study using Electrocardiogram (ECG) feature is discussed in the article to monitor health in real time.
